# Social and cultural dimensions of hygiene in Cambodian health care facilities

**DOI:** 10.1186/1471-2458-11-83

**Published:** 2011-02-07

**Authors:** Pascale Hancart-Petitet, Céline Dumas, Anne-Laure Faurand-Tournaire, Alice Desclaux, Sirenda Vong

**Affiliations:** 1 Institut Pasteur du Cambodge, Phnom Penh Cambodia; 2 Groupe de Recherche Cultures, Santé, Sociétés, Université Paul Cézanne d'Aix Marseille, France; 3 Institut de Recherche et Developpement (UMIR 233 « VIH/Sida et maladies associées »), Dakar, Sénégal

## Abstract

**Background:**

The frequency of bloodborne pathogen healthcare-associated infections is thought to be high in developing Southeast Asian Countries. The underlying social-cultural logics contributing to the risks of transmission are rarely studied. This report provides some insights on the social and cultural factors that shape hygiene practices in Cambodian health care settings.

**Methods:**

We conducted qualitative surveys in various public and private health facilities in Phnom Penh, the capital city and in provinces. We observed and interviewed 319 participants, health care workers and patients, regarding hygiene practices and social relationships amongst the health care staff and with patients. We also examined the local perceptions of hygiene, their impact on the relationships between the health care staff and patients, and perceptions of transmission risks. Data collection stem from face to face semi-structured and open-ended interviews and focus group discussions with various health care staffs (i.e. cleaners, nurses, midwives and medical doctors) and with patients who attended the study health facilities.

**Results:**

Overall responses and observations indicated that hygiene practices were burdened by the lack of adequate materials and equipements. In addition, many other factors were identified to influence and distort hygiene practices which include (1) informal and formal social rapports in hospitals, (2) major infection control roles played by the cleaners in absence of professional acknowledgment. Moreover, hygiene practices are commonly seen as an unessential matter to be devoted to low-ranking staff.

**Conclusion:**

Our anthropological findings illustrate the importance of comprehensive understanding of hygiene practices; they need to be considered when designing interventions to improve infection control practices in a Cambodian medical setting.

## Background

The frequency of bloodborne pathogens transmission in health settings has been considered to be high in developing countries [1]. Despite many studies and surveys on infection control and transmission risks in medical settings, the underlying social and cultural logics contributing to this transmission are not well documented [2,3]. Besides epidemiological aspects, hospital hygiene is shaped by medical norms and social-cultural representations, and hygiene practices always take place within social relations [4-9].

In Cambodia, HIV prevalence was 0.9% among 15-49 year old adults in 2006 [10]. The burden of hepatitis B virus (HBV) and hepatitis C virus (HCV) infections is considered high. Caruana *& al *[11] estimated that the prevalence amongst adult Cambodian migrants in Australia is 8% for both HBV and HCV. In Cambodia, several specific studies [12,13] suggest that HBV prevalence is around 8-9% and HCV prevalence is ~1-2% (Institut Pasteur Cambodia's unpublished data). It is likely that HBV transmission has been linked to healthcare-associated infection since high frequency of therapeutic injections combined with unsafe injection practices still occur in hospitals [14,15] or in the private sector [16,17].

From 2006 to 2009, anthropological studies were conducted in different health settings in Cambodia to identify social and cultural determinants of hygiene practices - particularly those related to blood borne pathogen transmission (Project ANRS 12102): (1) national reference hospitals which included a maternity and a general hospital in Phnom Penh; (2) public health centers in the province including some that were supported by a non-governmental health organization (NGO); and (3) private maternal and child clinics that belonged to the private sector. The latter is recognized to attract most of Cambodians when they first seek care [18]. The public sector is unregulated in which lay health professionals can practice at will and drugs are delivered without prescriptions.

This report summarizes the common findings of these anthropological studies, examining (1) local discourses and practices regarding hygiene in a health facility, (2) social relationships between health care workers and patients; and (3) their representations of hygiene, and their perceptions of infectious risks.

## Methods

### Data collection using qualitative methods

Qualitative research explores people's subjective understandings of their everyday lives. The methods used in these studies include direct observation, interviews in the form of conversation/talk to collect data about people's views and experiences [19]. Interviews were individual or *via *focus group discussions (group interviews). The investigation was led by researchers whose expertise was on anthropology with professional backgrounds in health (medical doctor, midwife and pharmacist) and their local assistants who were experienced in social sciences-based interviews. Discussion guides were developed to facilitate the flow of conversation using open-ended questions and semi-structured interviews [20]. The researchers spent several days, weeks or months in various health facilities approaching various people from different backgrounds. The aim was to transform the interview into a situation of ordinary interaction, designed to minimize the artificiality and the bias produced by a formal interview. It requires prolonged interaction between the researcher and local people to produce knowledge in situ and to document the interviewees' perspective and usual practices, and related meanings [21]. Conversations commonly lasted thirty minutes to three hours. Main participants were interviewed several times. The main issues discussed related to the professionals' experiences and the patients' experiences in health settings related to hygiene, perceptions of virus and microbes, availability and use of material and equipment, training, knowledge, and practices related to hygiene.

### Data analysis

The transcription of notes or recorded speech using radiotapes was done immediately following the interviews between the researcher and the local assistant. We analysed by categories of meaning and processed according to the following questions: which terms are used by the interviewees to describe the investigated issues ? Which themes may be identified by the researcher even if the interviewees did not consider them ?[22].

### Identification of interviewees

The researchers contacted resources persons (i.e. individuals responsible for non-governmental organizations or national institutions such public health facilities) who provided names of potential interviewees. Other interviewees were then selected on the basis of respondent-driven sampling called in anthropology the "network approach". Each interview led to new contacts suggested directly or indirectly during the conversation.

### Study sites

We selected private and public health facilities that were primarily used by poor population: one general hospital, the largest maternity, two health centers and two midwives' private clinics in Phnom Penh. We collected information regarding the organization of the hospital wards, availability of equipements and consummables, social rapports between caregivers and patients, and overall social conditions that led to at-risk hygiene practices. In addition, we interviewed some health centers' staff, patients traditional healers and traditional birth attendants in Prey Veng, Svay Rieng and Kampong Chhnang provinces.

### Target population

We penetrated 14 networks that led to interviewing 319 individuals including 40 medical doctors, 22 midwifes, 22 nurses, 32 cleaners, 4 pharmacists, 4 traditional healers and 5 traditional birth attendants, 20 social workers and 170 patients (including 15 people living with HIV and 12 sex workers. We also conducted focus group discussions with women attending reproductive health counselling sessions (2) and with medical students (3). Besides, we collected information related to hospital organization, and observed medical practices such as injections (39), infusions (19), pleural punctures (7), bronchoscopies (5), deliveries (27), post abortion care (4), abortions (1), Intra Uterine Diaphragme insertions (3). We also observed how sterilization, cleaning practices and laundry were processed.

### Ethical considerations

To encourage an open expression of opinions, only verbal consents were obtained and confidentiality and anonymity of all data were insured for participants. Studies were approved both by the Cambodian National Ethics Committee for Health Research and the French Agency for AIDS & HIV research's Institutional Review board.

## Results

### Working conditions

Great disparity amongst health care settings was observed. No cleaners were appointed in primary health care centers. The number of cleaners appointed in provincial hospital was scarce comparatively to capital hospitals. Reference hospitals were given an amount of materials and resources that were reported as sufficient by caregivers and cleaners. As scholars observed previously [23,24], Provincial hospitals were receiving less and the Primary Health Centers were the poorest health institutions. Interviewees generally reported that materials and equipments were not distributed on a regular basis and were insufficient to meet the requirements of the Ministry of Health's package of health care activities. For instance, a maternity in Phnom Penh city which is attended by more than 100 patients per day, was only supplied with the following volumes per month: 5 liters of bleach, 5 kg washing powder, 5 bottles of perfumed cleaning product, 4 brooms. Health centers' staff reported frequent shortage periods and the hospital staff often complained that equipments were out of order or not repaired.

Some health care practices may have led to on-going "penny pinching" practices. For example, cleaning products were diluted and many expired products were still used. To limit the use of injection equipments, caregivers were observed using the same syringe and needle on the same patient but for different drugs, even if the injection equipments were available. Midwives reported not using the same syringe and needle for different patients, but allowed themselves using the same ones to inject oxytocine and then lidocaine for one delivering woman.

We visited private midwives' clinics that were commonly attended by many deprived women we interviewed. All were located in precarious locations. Reproductive health practices that were observed included Depoprovera injection, intra uterine diaphragme and abortions. The use of old or already used equipements was common (i.e. old and corroded scissors, clamp and curette, multiple time used abortion kit containing a 500 ml syringe and a cannula). Sterilization procedures were limited in absence of autoclaves. Instruments were at best cleaned with water and soap and sometimes boiled or fambered with alcohol.

### Hygiene practices driven by individual perceptions

Many patients and caregivers perceived hygiene as related to the notion of clean and nice. In khmer language clean and nice bears the same word "*saat"*. Except among the medical staff, the rest of the interviewees rarely mentioned asepsis and sterilization. These perceptions led to some hygiene practices that were implemented by the cleaners (or worker *kamacors*) in order to meet aesthetic standards. For example, cleaners' reports related to clothes cleaning pointed out how bleach was used in order to remove blood stains. However, cleaners insisted that bleach dosage for this task is very sensitive: they will be reprimanded by their supervisors if, due to extra use of bleach, clothes are bleached. Patients' perceptions of hygiene while attending a health structure are also constructed according to aesthetic and order criteria. A place is considered clean if it is *"well-lit and when no spider webs are seen"*.

Levels of staff training in hygiene related practices were very unequal amongst the caregivers of various health settings. Training is mainly given orally during the first days on the job. Furthermore, access to training and to knowledge is related to hierarchical position, initial training and seniority in the health institution. Recently trained individuals were often reluctant to share their newly acquired knowledge with others.

The hospital staff indicated that hygiene protocols are often useless, hence not used. In many hospital wards, the cleaning service was done parcimoniously and in a random location throughout the morning: a short and localized sweep thereby, passing the mop in two rooms, or wiping on a stain of blood. We never observed systematic and complete washing/cleaning as recommended by the Protocol developed by the Department of Infection Control. For instance, the cleaners had to clean - squatted on the floor - clothes soiled by infants' feces, women's blood, waste from deliveries or gynecological interventions. Clothes were then cleaned by trampling underfoot (with plastic boots) and with jets of water. Soiled water is finally wiped out into the gutter.

The infection control committee protocol based on WHO norms [25] which gives various rules and pictures related to the hospital and material cleaning, hand washing, waste management, may be available in hospital but was never used during our observation periods. To manage the daily activity with various restrictions in terms of material and products supplied by the hierarchy, cleaners implement their own recipes for products uses. For example, they mixed washing powder, bleach, and a Thai perfumed cleaning product with water in an old plastic bottle. According to them, this mixed product is useful to kill *merouk *(microbes). In addition, it was said that washing powder cleans and Thai product removes bad smell.

In public referral hospital and private clinic managed by NGOs, hygiene procedures did exist and were implemented. However, no hygiene protocols were implemented for reproductive health care in a primary health center and low standard private clinic. Moreover, the lack of medical material drove caregivers into applying hygiene procedures according various logics. They reckoned their made-up methods are better suited to their daily needs and local constraints. Thus, hygiene protocols were either revised to be less costly (e.g. when a product is diluted) or they were transposed from the domestic area. For example, chlorexidine diluted in water, and normally used in order to decontaminate instruments was replaced by washing powder or stone of alum. In some cases, a small domestic electric oven is used as an autoclave: the material is "cooked" for 30 minutes at 100°C. One hospital-based doctor explained how the sterilization procedures applied by the staff were similar to cooking food. He told us:

*"Do you know how to cook the steamed fish? At the hospital, the staff uses the same technique as for fish. Instruments are put in a water pot, and then cooked*. *When the water is boiling, that's ready!!!"*

### Cleaners as key actors in hygiene

Most cleaners or *kamacor, worker *in Khmer language - who are at the bottom of hospital hierarchy, had not received proper training on hygiene and waste management and yet they were responsible for cleaning, disinfection, sterilization, waste management (contaminated or not) in the hospitals.

In the public hospital, the cleaners were reported as the patients' privileged interlocutors, particularly because of their similar social and economical status. So, they appeared to relay hospital rules and procedures (norms) to patients or their relatives. They often gave advices to the patient about health, helped them for administrative matters. They guided patients' relatives in various tasks which included giving suggestions on how to clean and arrange the space occupied by their patients in the hospitalization ward. For example, the cleaners would recommend to patients' relatives not to hang out washed clothes inside the hospital rooms. Furthermore, cleaners gave specific advices on body hygiene. We observed in the maternity in Phnom Penh that some cleaners explained to pregnant women as to how to proceed regarding personal hygiene before medical examination in the delivery room. One worker explained:

"I said to women to wash their body and to change their clothes. If they have a bad smell the doctor wouldn't examine them and he will not take care of them properly."

Hospital social organization was strongly based on social hierarchy which had various effects when implementing hygiene practices. We observed various cases of "strategy of task shifting": caregivers (doctors or nurses) gave the cleaners various infection control tasks that were devoted to them; for example we observed in one maternity the cleaners cleaning the places and materials with blood and dejections or pulling out a catheter for IV infusion. They were made responsible for needles waste boxes and could also help the mother to breastfeed the newborn. The workers were also involved in preparing materials for sterilization and had to perform various activities that were normally devoted to doctors or nurses such as removing an intravenous infusion catheter or assisting a surgeon during a surgical procedure. As a worker (woman) said:

"At the operating room, we wash the blood on the table, materials and fold laundry. All of this is the nurses and doctors' job; they have to do this! But we're doing it because we have to keep good rapport with them and help them."

### Health professionals' perceptions of risks

Being a caregiver is mostly seen as a hazardous activity. The risk of occupational transmission is often mentioned, and it is linked to blood and microbes perception. *"When we are injured by used needles we fear to die"*, said one worker in Phnom Penh. Besides, the fear of microbes was always mentioned by health workers. But they made a distinction between risks for contagiousness and dangerousness. While the acid- fast-bacilli or the influenza viruses were known to be contagious, HIV and the hepatitis viruses were both considered as the most contagious and dangerous.

In addition, caregivers felt isolated when facing a potential contamination. They must handle all the protective practices with limited stocks of disposable materials. They did not always have gloves to wear, and they must bear the costs of testing for HBV infection. Despite free access to HIV post exposure prophylaxis in Cambodia through a National HIV Control Program, some cleaners reported to have paid for the treatment. Furthermore, cleaners in some provincial health facilities had no acccess to it. In addition, caregivers cope with personal protection on their own. There was no collective management of risks (no effective protocol for example). This led to a lack of collective responsibility in case of professional contamination. For example, while medical institutions did not provide safe working conditions, denunciation of these institutions was never mentioned or even considered by hospital staff when occupational contamination occurred. Professional identity, membership in the medical corporation, did not appear to provide a sense of protection and caregiver's affiliation seemed to be lacking.

Other places showed a multiplication of protective materials: caregivers in the delivery room wear sterile gloves under their thick rubber gloves when attending some patients. But the general rule was, above all, that these protective measures were made for health professionals, not for patients. These "ego protective" practices prevailed: for example, we observed that hand washing was systematic before living the workplace, but not during working time.

### Midwives facing infection risks

Midwives reported a rigid social and professional hierarchy in which they are not allowed to have a say if they observed that doctors do not comply with the aspesis rules. In addition, the midwives were convinced that their practices were adequate, or were the best ones, because they were working in the main referral hospital. They had no doubt that midwives from Phnom Penh were better trained and better valued than their provincial colleagues although we observed many technical mistakes (sterile gloves put on a non sterile table during delivery, sterile gloves put on top of non sterile gloves, facial mask reused all day long and stored in a pocket, same syringe reused for the same woman with oxytocin first and xylocain after).

Most midwives implemented "ego protective" practices; they kept the same pair of gloves throughout the day to protect themselves from the potentially dangerous environment. An additional and same pair of sterile gloves tigthened with an adhesive around the wrist would be used to bath different newborns.

While attending delivery, midwives reported a constant fear of being infected by HIV or hepatitis viruses. This fear stem from regular and common handling of biological products like blood or amniotic fluid. This led to social distancing between midwives and pregnant women which was exacerbated by the fact that most patients belonged to lower social class. In practice, the caregiver scarcely touched the patient fearing the contact with the dirt and sweat of a stranger. It was observed many times that midwives finger- pointed openly dirts on patients' bodies which suggests that norms for patient-healer relationships rely on a physical distance.

### Patients' perceptions of infection risks

Patients reported fearing contamination or infection particularly while seeking care in small private clinics run by biomedical caregivers or not always properly trained care providers. However, their access to safe practices was limited by the costs of such services. As an example, here is a comment of a married woman who had several abortions in various low-resource health settings.

"it was at the health workers' houses, as you know, and the hygiene isn't the same as in the hospitals. But if you go to the hospital, it's expensive. You can't afford to pay!"

Issues related to hygiene were also framed by power relationships between patients and caregivers. For example, one woman who had an abortion in a public institution told us:

« I couldn't know if the material they used was sterilized or not. Yes I feared the infection but I didn't dare to ask, I feared much more the anger of the midwife! »

## Discussion

Our findings were unique in shedding light on issues related to infection control in Cambodia. They raised social and cultural issues which had repercussions that may find echoes in countries of similar backgrounds. These perceptions and issues were exacerbated in settings where resources are limited.

Our main results showed that hygiene practices are shaped firstly by the lack of disposable materials and equipments. These chronic equipment shortages in addition to the lack of knowledge about appropriate hygiene practices observed at various levels have led to practices built on more empirical than professional bases. The technical content of practices relied therefore on idiosyncratic perceptions and appears to have been made to meet aesthetic criteria and demands of hierarchy rather than informed approaches.

Another important finding was the crucial role in hospital hygiene that incombed to the cleaners, also known as *kamacor*. Their role is not recognized by those who « own knowledge », i.e. midwives and doctors. Actually, these cleaners hold the link between medical departments, as they work in all of them in order to collect dirty clothes and waste. They are also the links between caregivers in the hospital organization. In spite of these charges, the cleaners have the lowest salaries and remain at the lowest level of the hospital hierarchy. Their training is also neglected and in some cases, these cleaners are easy targets for reprimands. Thus hygiene practices, as the main task of cleaners, remain often invisible as those tasks have a very low symbolic and social capital. Such a relegation of workers dealing with hygiene in hospital settings is not specific to Cambodia and has been described also in developed countries [26].

The reluctance of medical and nursing staff in "touching" patients and their tendency to choose "noble" tasks and delegate others to lower level staff is amplified by social hierarchy between medical staff and patients as well as between cleaners and medical staff. Those findings raise several questions related to the construction of caregivers' professional roles, the place given to empathy in caregivers/patient relation and meanings of body contacts in the overall social system [27]. It refers to the whole historical and social construction of biomedical system in Cambodia [28,29,24].

Our study had limitations. Firstly, the study was constructed with long durations of immersion "in the field" to mitigate observation biases [30,31] and using semi-structured and open-ended interviews which made it difficult to categorize and enter data in a database to be analyzed in a quantitative way. Our approach has however brought about new understanding for a situation that needs to be further explored. Secondly, we chose to visit health facilities that were not well equipped; however, we reckon that these types of health facilities we visited were attended by the majority of the population as 68% of the population lives with less that two dollars a day [32].

Our field data raise many issues that may be considered for improvement in hygiene or for futher investigation. Besides general recommendations such as improving training on infection control and adapting it to locally relevant topics, besides supplying adequately consummables and equipments to ensure appropiate hygiene practices and a sense of safety among health workers, we also recommend to insist on the central role played by the cleaners in hospital hygiene. It would seem relevant to increase the status of these workers and make sure that their role in hygiene management becomes visible. Moreover, the cleaners are ideal candidates for future training programs related to hygiene.

Finally, the social distance and the power relation maintained by caregivers while providing care to patients have various impacts on medical practices. Some of those practices have consequences for hygiene management. So, individual equipments used by caregivers respond to a logic of "ego-protection." This logic results in omitting or neglecting patient's risk and the issue of healthcare associated infections is often considered as secondary to caregivers' concerns. Hygiene practices enhancement could be improved through teaching work ethics during medical studies and in encouraging patients' empowerment and dialogue between caregivers and patients [33].

## Conclusion

Various anthropological studies conducted in developing countries have documented the social issues at stake in medical settings that impact on patients care. For example, enlightening the social determinants of maternal mortality in a West African hospital [34] or examining the various constraints that shape patients observance of Antiretroviral treatment in Senegal [35] has been very useful to adjust medical program activities to specific contexts. Similarly, our study has underscored the needs of an integrated anthropological approach in analyzing the social construction of medical practices and risks of infection transmission particularly in a country of limited resources and poorly trained staff. Without these social considerations many hospital infection control programs may fail to meet and sustain their objectives.

## Competing interests

The authors declare that they have no competing interests.

## Authors' contributions

AD designed and led the project. SV was the corresponding scientist for the project at the Institut Pasteur - Cambodia. PHP, CD and ALFT conducted the study, collected, analyzed and interpreted the data. PHP wrote the manuscript including the social sciences aspects and SV wrote the public health aspects. AD and SV completed the version to be published. All authors read and approved the final manuscript.

## Authors' information

Alice Desclaux, MD, PhD, Prof in Medical anthropology, Groupe de Recherche Culture Sante Société (GReCSS), Université Paul Cézanne d'Aix Marseille (UPCAM) and Institut de Recherche et Developpement (UMIR 233)

Sirenda Vong, MD, MSc, Head of Epidemiology and Public Health Unit, Institut Pasteur - Cambodia

Pascale Hancart Petitet, PhD, (Research Fellow, Epidemiology and Public Health Unit, Institut Pasteur -Cambodia, GReCSS/UPCAM),

Céline Dumas, Pharm. D, MA (Pharm. D., Master Degree in Anthropology, GReCSS/UPCAM)

Anne-Laure Faurand-Tournaire, MD, MA (Master Degree in Anthropology, GReCSS/UPCAM)

## Pre-publication history

The pre-publication history for this paper can be accessed here:

http://www.biomedcentral.com/1471-2458/11/83/prepub
